# *Let-7* miRNA-binding site polymorphism in the *KRAS* 3^′^UTR; colorectal cancer screening population prevalence and influence on clinical outcome in patients with metastatic colorectal cancer treated with 5-fluorouracil and oxaliplatin +/− cetuximab

**DOI:** 10.1186/1471-2407-12-534

**Published:** 2012-11-20

**Authors:** Janne B Kjersem, Tone Ikdahl, Tormod Guren, Eva Skovlund, Halfdan Sorbye, Julian Hamfjord, Per Pfeiffer, Bengt Glimelius, Christian Kersten, Hiroko Solvang, Kjell M Tveit, Elin H Kure

**Affiliations:** 1 Department of Genetics, Institute for Cancer Research, Oslo University Hospital, Oslo, Norway; 2 Department of Oncology, Oslo University Hospital, Oslo, Norway; 3 School of Pharmacy, University of Oslo and the Norwegian Institute of Public Health, Oslo, Norway; 4 Department of Oncology, Haukeland University Hospital, Bergen, Norway; 5 Department of Oncology, Odense University Hospital, Odense, Denmark; 6 Department of Radiology, Oncology and Radiation Science, Uppsala University, Uppsala, Sweden; 7 Department of Oncology and Pathology, Karolinska Institutet, Stockholm, Sweden; 8 Center for Cancer Treatment, Southern Hospital Trust, Kristiansand, Norway

**Keywords:** Colorectal cancer, LCS6, MiRNA polymorphism, Cetuximab, Oxaliplatin, 5-fluorouracil

## Abstract

**Background:**

Recent studies have reported associations between a variant allele in a *let-7* microRNA complementary site (LCS6) within the 3^′^untranslated region (3^′^UTR) of *KRAS* (rs61764370) and clinical outcome in metastatic colorectal cancer (mCRC) patients receiving cetuximab. The variant allele has also been associated with increased cancer risk. We aimed to reveal the incidence of the variant allele in a colorectal cancer screening population and to investigate the clinical relevance of the variant allele in mCRC patients treated with 1^st^ line Nordic FLOX (bolus 5-fluorouracil/folinic acid and oxaliplatin) +/− cetuximab.

**Methods:**

The feasibility of the variant allele as a risk factor for CRC was investigated by comparing the LCS6 gene frequencies in 197 CRC patients, 1060 individuals with colorectal polyps, and 358 healthy controls. The relationship between clinical outcome and LCS6 genotype was analyzed in 180 mCRC patients receiving Nordic FLOX and 355 patients receiving Nordic FLOX + cetuximab in the NORDIC-VII trial (NCT00145314).

**Results:**

LCS6 frequencies did not vary between CRC patients (23%), individuals with polyps (20%), and healthy controls (20%) (*P* = 0.50). No statistically significant differences were demonstrated in the NORDIC-VII cohort even if numerically increased progression-free survival (PFS) and overall survival (OS) were found in patients with the LCS6 variant allele (8.5 (95% CI: 7.3-9.7 months) versus 7.8 months (95% CI: 7.4-8.3 months), *P* = 0.16 and 23.5 (95% CI: 21.6-25.4 months) versus 19.5 months (95% CI: 17.8-21.2 months), *P* = 0.31, respectively). Addition of cetuximab seemed to improve response rate more in variant carriers than in wild-type carriers (from 35% to 57% versus 44% to 47%), however the difference was not statistically significant (interaction *P* = 0.16).

**Conclusions:**

The LCS6 variant allele does not seem to be a risk factor for development of colorectal polyps or CRC. No statistically significant effect of the LCS6 variant allele on response rate, PFS or OS was found in mCRC patients treated with 1^st^ line Nordic FLOX +/− cetuximab.

## Background

The prognosis for patients with metastatic colorectal cancer (mCRC) included in clinical trials has increased from approximately 12 months with 5-fluorouracil monotherapy to 20–24 months with the addition of newer chemotherapeutic agents and targeted drugs [1]. Cetuximab and panitumumab, two monoclonal antibodies (moAb) targeting the epidermal growth factor receptor (EGFR), have proven to be effective in combination with chemotherapy or as single agents [2-7]. *KRAS* mutation is a negative predictive marker for response to EGFR-targeted therapy and clinical benefit seems to be restricted to patients with *KRAS* wild-type tumors [2-5,8,9]. In the recent NORDIC-VII study, however, we did not find an improved outcome of adding cetuximab to first-line oxaliplatin-based chemotherapy in *KRAS* wild-type patients [10]. Similar results were found by the COIN trial [11]. The results of these trials demonstrate the necessity to explore and validate new biomarkers to improve the selection of patients who are likely to benefit from cetuximab treatment [12]. It was recently reported that copy number aberrations (CNA) may provide additional information to mutation status and their use may potentially further improve the selection of mCRC patients for EGFR-targeted therapy [13]. Mekenkamp *et al.* demonstrated that *KRAS* copy number loss was associated with good response in both *KRAS* wild-type and *KRAS* mutated mCRC patients treated with a cetuximab-containing first-line regimen [13].*KRAS* copy number gains were associated with poor progression-free survival (PFS) in *KRAS* wild-type mCRC patients given the same treatment [13].

MicroRNAs (miRNAs) are a class of highly conserved 22-nucleotides single-stranded RNAs that can act as trans-acting factors that suppress translation or induce messenger RNA (mRNA) degradation of target genes [14]. They are global gene regulators implicated in virtually all cancer types studied, where they can function as oncogenes or tumor suppressors [15]. A number of miRNAs have been reported to be involved in CRC development and *KRAS* regulation, and these may influence the effect of EGFR-targeted therapy [13,16,17]. An important miRNA in CRC seems to be *miR-143,* which, has been described to be downregulated in CRC [18] and to inhibit the translation of *KRAS* mRNA, thereby altering RAS signaling and inhibiting tumor cell growth [19]. Pichler *et al.* found that low *miR-143* expression was an independent negative prognostic factor for cancer specific survival in mCRC, and they reported a decreased PFS in *KRAS* wild-type mCRC patients treated with EGFR-targeted agents [16].

The *let-7* family of miRNAs plays an important role in many malignant tumors where they mainly function as tumor suppressors. Downregulation of *let-7* family members is observed in multiple carcinomas [20], including colon cancer [21]. RAS expression was decreased in colon cancer cell lines after transfection of *let-7a-1* miRNA precursor suggesting that *let-7* is involved in regulating colon cancer cell growth [22]. *Let-7* miRNAs downregulate RAS after binding to specific sites in the 3^′^ untranslated region (3^′^-UTR) of the *KRAS* mRNA [23]. A functional single nucleotide polymorphism has been characterized in the *let-7* complementary site (LCS6) in the *KRAS* 3^′^-UTR mRNA leading to increased expression of *KRAS in vitro* and lower *let-7* levels *in vivo*[24].

The LCS6 variant allele is associated with increased risk of non-small cell lung cancer (NSCLC) in moderate smokers [24], triple-negative breast cancer in premenopausal women [25], and ovarian cancer in BRCA negative women from hereditary breast and ovarian cancer syndrome families [26]. Moreover, the LCS6 variant allele is enriched in BRCA negative double primary breast and ovarian cancer patients [27]. One study failed to find an association between the LCS6 variant allele and sporadic or familial ovarian cancer risk [28]. A recent study confirms the importance of the LCS6 variant allele in postmenopausal ovarian cancer patients and demonstrates that it is a biomarker of poor outcome in this disease, probably due to platinum resistance [29]. The LCS6 variant allele is also associated with reduced survival in oral cancer [30]. On the contrary, Smits *et al.* found that early-stage CRC patients with the LCS6 variant allele had better outcome [31], whereas Ryan *et al.* recently reported the LCS6 variant allele to be associated with reduced risk of mortality in late-stage CRC [32].

We determined the frequency of the LCS6 variant allele in a Norwegian case–control study, the KAM cohort, consisting of CRC patients, individuals with polyps in the large intestine, and healthy controls, to investigate the feasibility of the variant allele as a risk factor for CRC development.

Three studies have reported conflicting results regarding the relationship between the LCS6 variant allele and clinical outcome in mCRC patients receiving cetuximab [33-35]. In this work we have studied if the LCS6 variant allele was associated with clinical outcome in NORDIC-VII, a randomized phase III trial where Nordic FLOX (bolus 5-fluorouracil/folinic acid and oxaliplatin) was given with or without cetuximab as first-line treatment in mCRC [10].

## Methods

### NORDIC-VII

In the NORDIC-VII trial (NCT 00145314), patients with mCRC were randomized to receive first-line standard Nordic FLOX (bolus 5-fluorouracil/folinic acid and oxaliplatin) (arm A), cetuximab and Nordic FLOX (arm B), or cetuximab combined with intermittent Nordic FLOX (arm C) [10]. Primary endpoint was progression-free survival (PFS), overall survival (OS) and response rate were secondary endpoints. Mutation analysis of *KRAS* and *BRAF* were performed. Cetuximab did not add significant benefit to Nordic FLOX and *KRAS* mutation was not predictive for cetuximab effect.

DNA from a total of 535 of the 566 patients in the intention to treat population was evaluable for LCS6 genotyping. Of the 31 samples that were not evaluable five were due to low DNA concentration, seven to undetermined results, and 19 samples were not available. There were 180 patients in arm A and 355 patients in arms B and C evaluable for response and survival analyses. Response status was evaluated according to the RECIST version 1.0 criteria and was assigned to patients with complete or partial remission with changes in tumor measurements confirmed by repeat studies performed no less than 4 weeks after the criteria for response were first met (minimum interval of 8 weeks – 4 cycles) [36]. The study was approved by the national ethics committees and governmental authorities in each country and was conducted in accordance with the Declaration of Helsinki. All patients provided written informed consent.

### KAM cohort

The KAM cohort is based on the screening group of the Norwegian Colorectal Cancer Prevention study (The NORCCAP study) (NCT00119912) [37] and CRC cases (not evaluated for metastases) from Telemark Hospital and Oslo University Hospital. Evaluable blood samples for LCS6 genotyping were obtained from 197 CRC patients, 1060 individuals with polyps in the large intestine, and 358 healthy controls. All participants in the KAM study provided written informed consent. The project was approved by the Ethics Committee REK South-East, Norway (Regional komite for medisinsk og helsefaglig forskningsetikk Sør-Øst).

### LCS6 genotyping

The single nucleotide variant allele rs61764370 is located in the *let-7* complementary site 6 (LCS6) of the *KRAS* 3^′^ UTR. The LCS6 genotype was determined using an in-house TaqMan® allelic discrimination assay on a Sequence Detection System ABI 7500. Forward and reverse primer sequences are TGTGCCACTACACTCAACTAATTTTTG and TGGTAGGCACTCAATAAATATTTGCT respectively. Probes were labeled VIC (wild-type) and 6FAM (variant type) with sequences VIC-TGACCTCAAGTGATTCA-MGB and 6FAM-AAGTGATGCACCCACCTT-MGB (Applied Biosystems). A BLAST search of primer and probe sequences was performed with adequate specificity for the locus of interest. The assay was performed in 10 μL reactions containing 1 x Mastermix, 200 nM of each probe, 900 nM of each primer, and 20–40 ng of genomic DNA extracted from peripheral blood. Cycling conditions were according to the TaqMan® Genotyping Assay Protocol: 50°C for 2 min, 95°C for 10 min, and 40 cycles of 95°C for 15 s and 60°C for 1 min. Genotype calling was performed semi-automated using the Sequence Detection Software v1.3 (Applied Biosystems). The analyses were performed in duplicate.

### Statistical analyses

As a consequence of the low frequency of the homozygous LCS6 G-allele, the G/T and G/G genotypes were collapsed in the analyses. For the prognostic analyses all three arms (arms A, B and C) were analyzed together. For the predictive analyses arm A was compared to arms B and C together. The association of the LCS6 genotype, *KRAS* and *BRAF* mutation status was analyzed by the chi-square test. The association between the LCS6 genotype and tumor response was analyzed by binary logistic regression for the prognostic and predictive analyses. OS and PFS times were estimated using the Kaplan-Meier method. The associations of the LCS6 genotype and PFS and OS were analyzed by Cox’s proportional hazard regression model. The value of LCS6 as a predictive marker of cetuximab effect was analyzed by including an interaction term in the models. The distribution of genotypes in the NORDIC-VII study and the KAM cohort were tested for Hardy-Weinberg equilibrium [38]. The distribution of the LCS6 genotype in the CRC patients, the individuals with polyps and the healthy controls from the KAM cohort was analyzed by the chi-square test. *P* < 0.05 was considered statistically significant. All p-values were two-sided. The statistical analyses were performed using Statistical Package for Social Sciences, version 18.0. (SPSS Chicago, IL).

## Results

### LCS6 variant allele and CRC risk

The frequency of the LCS6 variant allele in the KAM cohort was 20% in healthy controls (70/358; 2 homozygous and 68 heterozygous), 20% in individuals with polyps (209/1060; 16 homozygous and 193 heterozygous), and 23% in CRC patients (46/197; 1 homozygous, 45 heterozygous) (*P* = 0.5) (Table 1). The distribution of genotypes was in Hardy-Weinberg equilibrium (*P* = 0.34, 0.19, and 0.22 for healthy controls, individuals with polyps, and CRC patients, respectively).

**Table 1:** LCS6 genotype frequencies in controls, individuals with polyps and CRC patients

**Study population**	**Genotype**	**Frequency**
Controls	Wild-type (TT)	288/358 (80%)
	Variant (TG/GG)	70/358 (20%)
Individuals with polyps	Wild-type (TT)	851/1060 (80%)
	Variant (TG/GG)	209/1060 (20%)
CRC patients	Wild-type (TT)	151/197 (77%)
	Variant (TG/GG)	46/197 (23%)

### Characteristics of patients in the NORDIC-VII study

There were 84 (16%) carriers of the variant allele in the NORDIC-VII cohort. Three patients were homozygous, 81 heterozygous. The distribution of genotypes were in Hardy-Weinberg equilibrium (*P* = 0.75) and the distributions of the variant allele were similar in arms A, B, and C with frequencies of 14% (26/180), 18% (33/181), and 14% (25/174), respectively (Table 2). LCS6 status and *KRAS* mutation status were available from 471 patients, of which 183 (39%) were *KRAS* mutated. Patients with LCS6 wild-type had a *KRAS* mutation frequency of 40% (155/392) as compared to 35% (28/79) in patients with the variant allele. LCS6 status and *BRAF* mutation status were available from 435 patients, of which 53 (12%) were *BRAF* mutated. For patients with LCS6 wild-type, the frequency of *BRAF* mutation was 12% (45/361) as compared to 11% (8/74) in patients with the LCS6 variant allele. There was no significant association between *KRAS* or *BRAF* mutation status and LCS6 genotype (*P* = 0.53 and 0.71, respectively).

**Table 2:** Patient characteristics in the NORDIC-VII study

	**LCS6 wild-type (*N*=451)**			**LCS6 variant(*N*=84)**		
**Arm**	A	B	C	A	B	C
*N*	154	148	149	26	33	25
%	86	82	86	14	18	14
**Age, years**						
Median	60.4	60.8	63.7	63.8	60.6	62.3
Min	29.9	24.1	33.1	37.1	38.0	36.3
Max	74.8	74.4	74.9	74.7	73.7	74.0
**Sex (%)**						
Male	56	63	60	46	70	76
Female	44	37	40	54	30	24
**Location primary tumor (%)**						
Colon	55	53	66	65	61	52
Rectum	45	47	34	35	39	48

### Response rate and survival

**Figure 1:**
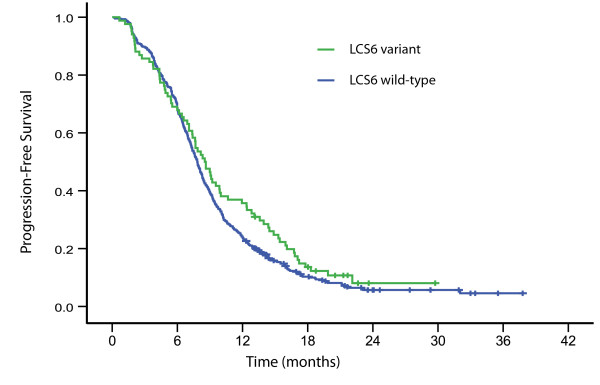
**Kaplan-Meier progression-free survival by LCS6 wild-type (*N *= 451) and LCS6 variant (*N *= 84), log rank *P *= 0.16.**

**Figure 2:**
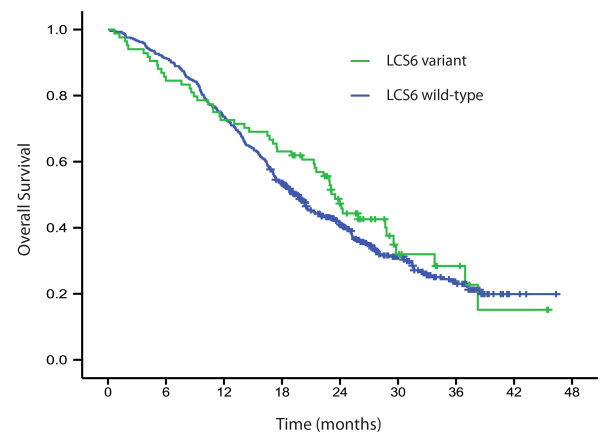
**Kaplan-Meier overall survival by LCS6 wild-type (*N*= 451) and LCS6 variant (*N*= 84), log rank*P*= 0.31.**

There was no significant difference in response rates in LCS6 wild-type of which 46% (208/451) responded, compared to 50% (42/84) in patients with the LCS6 variant allele (*P* = 0.55). Numerically increased median PFS and OS were found in patients with the LCS6 variant allele (8.5 months (95% CI: 7.3-9.7 months) and 23.5 months (95% CI: 21.6-25.4 months), respectively) compared to LCS6 wild-type (7.8 months (95% CI: 7.4-8.3 months) and 19.5 months (95% CI: 17.8-21.2 months), respectively), although not statistically significant (Log rank *P* = 0.16 and 0.31, respectively; Figures 1 and 2).

### Predictive analyses for benefit of cetuximab treatment

**Figure 3:**
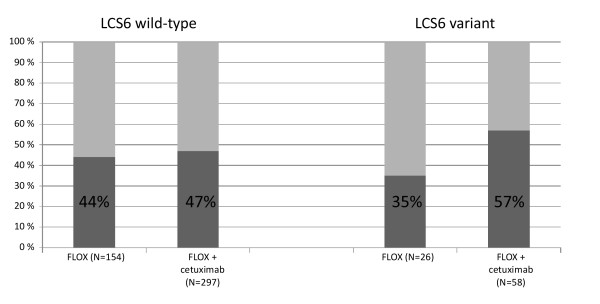
Confirmed response by LCS6 genotype and treatment.

Addition of cetuximab to FLOX seemed to improve treatment response more in patients with the LCS6 variant allele (35% in arm A versus 57% in arms B and C) than in wild-type carriers (44% in arm A versus 47% in arms B and C), but the difference was not statistically significant (interaction *P* = 0.16, Figure 3). Median PFS and OS were similar in arms B + C (8.5 months (95% CI: 7.0-10.0 months) and 23.5 months (95% CI: 19.9-27.1 months), respectively) as compared to arm A (7.7 months (95% CI: 3.8-11.6 months) and 22.3 months (95% CI: 13.2-31.4 months), respectively) in patients with the LCS6 variant allele (interaction *P* = 0.63 and 0.50, respectively, Table 3).

**Table 3:** Progression-free survival and overall survival by LCS6 genotype and treatment

**LCS6 genotype**	***N***	**Arm**	**Median months**	**95% CI**	**Interaction *P *value**
**Progression-free survival**					
Wild-type	154	A	7.9	7.2-8.5	0.63
	297	B+C	7.7	7.2-8.3	
Variant	26	A	7.7	3.8 – 11.6	
	58	B+C	8.5	7.0-10.0	
**Overall survival**					
Wild-type	154	A	20.4	16.5 – 24.2	0.50
	297	B+C	18.9	16.9 – 20.9	
Variant	26	A	22.3	13.2 – 31.4	
	58	B+C	23.5	19.9 – 27.1	

## Discussion

The frequency of the LCS6 variant allele in a Norwegian cohort was similar in healthy controls, individuals with polyps, and CRC patients. These results do not support the LCS6 variant allele as a candidate risk factor for development of colorectal polyps or CRC. The frequency of the LCS6 variant allele varies across geographic populations, with European populations exhibiting the variant allele most frequently [24,31,32,39,40]. The frequency of the LCS6 variant allele was 16% in 535 genotyped patients in the NORDIC-VII cohort, which is consistent with a recent study which analyzed 734 CRC cases from the Netherlands [31]. Interestingly, the frequency of the variant allele in mCRC patients in the NORDIC-VII cohort is significantly lower than in the CRC patients from the KAM cohort (*P* = 0.02). A possible explanation could be that the NORDIC-VII cohort consists of a more heterogeneous population from the Nordic countries compared to the KAM cohort, which consists of a homogenous Norwegian population. Another possible explanation could be that CRC patients with the LCS6 wild-type have a greater tendency to metastasize.

A recently published study found that early-stage CRC cases with the LCS6 variant had improved survival [31]. Another study reported that the LCS6 variant was associated with a reduced risk of mortality in late-stage CRC [32]. Numerically increased median PFS and OS were found in patients with the LCS6 variant allele when compared to LCS6 wild-type carriers, but the differences were not statistically significant at the 5% level and we cannot conclude that mCRC patients with the variant allele belong to a favorable prognostic group. Furthermore, the difference in treatment response of adding cetuximab was larger, albeit not statistically significant, in patients with the LCS6 variant. There was a non-significant trend of increased response rate for patients with the LCS6 variant allele when treated with 5-fluorouracil/oxaliplatin and cetuximab compared to 5-fluorouracil/oxaliplatin alone. The trend of numerically increased PFS, OS and response rate was also observed independent of *KRAS* mutation status and in *KRAS* and *BRAF* wild-type patients, but none of the findings proved statistically significant. Thus, any potential predictive effect of the LCS6 variant allele is likely to be too small to be demonstrated with the patient sample available from the NORDIC-VII study. The NORDIC-VII cohort has limitations for studies of biomarkers predictive of cetuximab effect, as cetuximab did not add significant benefit to the Nordic FLOX regimen. Also, the number of patients with the LCS6 variant allele is relatively small, and the analyses of LCS6 as a predictive marker thus have low power.

Zhang *et al.* demonstrated that of 67 *KRAS* wild-type mCRC patients, there was a higher response rate and a trend of longer PFS and OS in patients with LCS6 variant allele (*N*=12) compared to patients with LCS6 wild-type (*N*=55) when treated with cetuximab monotherapy [34]. Contrary, another study on 121 *BRAF* wild-type mCRC patients who underwent salvage cetuximab – irinotecan therapy, of which 58 were *KRAS* mutated, reported that patients with LCS6 variant allele (*N*=34) had shorter PFS and OS compared to LCS6 wild-type (*N*=87) [33]. Similar results were reported by Winder *et al.* who found mCRC patients with mutant *KRAS* and LCS6 variant allele to have shorter PFS when treated with irinotecan and cetuximab [35]. The conflicting results in these studies suggest that the chemotherapy backbone may play a role, and that the LCS6 variant allele have different predictive values in mCRC patients treated with cetuximab alone or in combination with 5-fluorouracil/oxaliplatin than in patients treated with cetuximab in combination with irinotecan [41,42].

Ragusa *et al.* demonstrated that cetuximab treatment induced miRNA transcriptome changes in drug-sensitive and drug-resistant CRC cell lines [17]. The set of differentially expressed miRNAs in the two cell lines (one sensitive and the other resistant) was almost entirely not overlapping. These data suggest that different responses to cetuximab are associated with different sets of miRNAs and thereby different molecular signaling. Interestingly, 67% of the differentially expressed miRNAs were involved in cancer, including CRC, whereas 19 miRNA targets had previously been reported to be involved in the cetuximab pathway and CRC. Based on their results, they suggest downregulation of *let-7b* and *let-7e* and the upregulation of miR-17* to be associated with cetuximab resistance [17]. Although these miRNAs were generated from cell studies, they illustrate that miRNAs may be promising predictive markers of cetuximab response to be further studied in mCRC patients.

We have only investigated one miRNA binding site polymorphism in this study, representing a small piece in a large puzzle of polymorphisms in the miRNA pathway. Future research on miRNA pathway polymorphisms as potential prognostic and/or predictive markers in mCRC should ideally include an integrated approach using bioinformatical tools combined with biological data to get a comprehensive understanding of the role and functions of miRNA polymorphisms and cetuximab response.

## Conclusions

The LCS6 variant allele does not seem to be a risk factor for development of colorectal polyps or CRC. No prognostic effect of the LCS6 variant allele was demonstrated in the NORDIC-VII cohort. No predictive effect of the LCS6 variant allele on response rate, PFS or OS when cetuximab is given in combination with 5-fluorouracil/oxaliplatin in the first-line treatment of mCRC patients could be proven. Although our study has a larger sample size than previously published studies, the sample size in the subgroup with the variant allele is still too low to obtain sufficient power to reliably conclude in subgroups based on treatment and genotype. Studies with larger sample size and with stratification of molecular subgroups and chemotherapy backbone are necessary to reveal if the LCS6 variant allele could be clinically useful when treating mCRC patients in the future.

## Competing interests

The authors declare that they have no competing interest.

## Authors' contributions

JBK performed the genotyping and prepared the first draft of the manuscript. JH established the method in the lab. KMT is the principal investigator of the NORDIC-VII study. KMT, TG, PP, BG, HS, and CK were responsible for the recruitment of patients, blood sampling and clinical data collection. EK was responsible for the biobanking. ES and HiS contributed with statistical advice. TG, TI, ES, JBK, HS, and EK were involved in the interpretation of the data and the conception of the manuscript. EK brought the idea and organized the study. All authors read and approved the final manuscript.

## Pre-publication history

The pre-publication history for this paper can be accessed here:

http://www.biomedcentral.com/1471-2407/12/534/prepub
